# SRPES and STM data for the model bimetallic Pd-In/HOPG catalysts: Effects of mild post-synthesis oxidative treatments

**DOI:** 10.1016/j.dib.2021.107626

**Published:** 2021-11-23

**Authors:** M.A. Panafidin, A.V. Bukhtiyarov, I.P. Prosvirin, I.A. Chetyrin, A.Yu. Klyushin, A. Knop-Gericke, N.S. Smirnova, P.V. Markov, I.S. Mashkovsky, Y.V. Zubavichus, A.Yu. Stakheev, V.I. Bukhtiyarov

**Affiliations:** a G. K. Boreskov Institute of Catalysis, Siberian Branch of the Russian Academy of Sciences, 630090 Novosibirsk, Russian Federation; b Fritz Haber Institute of the Max Planck Society, 14195 Berlin, Germany; c Helmholtz Center for Materials and Energy, 12489 Berlin, Germany; d Max Planck Institute for Chemical Energy Conversion, Department of Heterogeneous Reactions,45470 Mülheim an der Ruhr, Germany; e N. D. Zelinsky Institute of Organic Chemistry, Russian Academy of Sciences, 119991 Moscow, Russian Federation

**Keywords:** PdIn intermetallic compound, model catalyst, synchrotron radiation-based photoelectron spectroscopy, scanning tunneling microscopy

## Abstract

Post-synthesis treatment of bimetallic catalysts in different gas phases resulting in the adsorption-induced segregation is among promising approaches to enhance their activity not compromising selectivity towards a number of low-temperature reactions. Our recently published paper (M.A. Panafidin, A.V. Bukhtiyarov, I.P. Prosvirin, I.A. Chetyrin, A.Yu. Klyushin, A. Knop-Gericke, N.S. Smirnova, P.V. Markov, I.S. Mashkovsky, Y.V. Zubavichus, A.Yu. Stakheev, V.I. Bukhtiyarov, A mild post-synthesis oxidative treatment of Pd-In/HOPG bimetallic catalysts as a tool of their surface structure fine tuning. Appl. Surf. Sci.) reports on Pd-In intermetallic formation regularities and their evolution after storage in air as well as during treatment in oxygen at submillibar pressures. The current paper gives an extended representation of experimental *ex situ/in situ* synchrotron-based photoelectron spectroscopy (SRPES) and scanning tunnelling microscopy (STM) data used to derive scientific conclusions in the paper quoted above.

## Specifications Table

**Table:** 

Subject	Materials Science, Metals and Alloys
Specific subject area	Heterogeneous catalysis and physical characterization
Type of data	Table Image Graph Figure Chart
How the data were acquired	STM images were obtained on an RHK 7000 VT ultrahigh vacuum scanning tunneling microscope (RHK Technology, USA). XP spectra were measured with an in-lab photoelectron spectrometer (SPECS, Germany) equipped with a monochromator FOCUS 500 (AlKα radiation, hν = 1486.74 eV, 150 W) (preparation and characterization chambers). SRPES spectra were measured with a photoelectron spectrometer SPECS at the RGL-station at the Russian-German Laboratory (RGL) of the Helmholtz-Zentrum Berlin (HZB) equipped with an e-beam heating system (study of the Pd-In intermetallic compound (IMC) formation). NAP SRPES spectra were measured with a photoelectron spectrometer SPECS at the UE56/2 PGM-1 beamline of the HZB equipped with an infrared laser (O_2_-treatment).
Data format	Raw Analyzed Fitted
Description of data collection	XP spectra at preparation, characterization and IMC formation were recorded after each step in UHV (p ≤ 5 × 10^−9^ mbar) with lab-based and at RGL-synchrotron beamline setups, respectively. XP spectra during the O_2_-treatment were measured with a NAP-XPS setup (UE56/2 PGM-1) directly under the flow of 0.25 mbar O_2._ STM images were obtained in UHV in the constant current mode.
Data source location	G. K. Boreskov Institute of Catalysis, Siberian Branch of the Russian Academy of Sciences, 630090 Novosibirsk, Russian Federation Helmholtz Center for Materials and Energy, 12489 Berlin, Germany
Data accessibility	Repository name: Mendeley Data Data identification number: 10.17632/2h773jgvbm.2 Direct URL to data: https://data.mendeley.com/datasets/2h773jgvbm/2
Related research article	M.A. Panafidin, A.V. Bukhtiyarov, I.P. Prosvirin, I.A. Chetyrin, A. Yu Klyushin, A. Knop-Gericke, N.S. Smirnova, P.V. Markov, I.S. Mashkovsky, Y.V. Zubavichus, A.Y. Stakheev, V.I. Bukhtiyarov, A mild post-synthesis oxidative treatment of Pd-In/HOPG bimetallic catalysts as a tool of their surface structure fine tuning, Appl. Surf. Sci. 571 (2022) 151350. doi:10.1016/j.apsusc.2021.151350.

## Value of the Data

• The X-ray photoelectron spectroscopy dataset gives information about Pd and In depth distribution within Pd-In nanoparticles and its alteration during the storage of model Pd-In/HOPG samples in air, their subsequent stepwise heating in UHV up to 500 °C and directly during their treatment in 0.25 mbar O_2_.
• The raw and processed data may prove useful for surface scientists interested in the use of the O_2_-induced segregation as a tool for fine-tuning the surface structure of supported bimetallic catalysts to enhance their catalytic properties.
• The specific parameters of Pd and In deposition can be used by other researchers for the purposeful preparation of Pd- and In-containing model catalysts with desired stoichiometry and structure.
• Our analysis of raw experimental data with peak fittings of Pd3d and In3d core-level XP spectra into individual components can be useful for the identification of Pd and In chemical states in PdIn-based systems.

## 1 Data Description

**Fig. 1:**
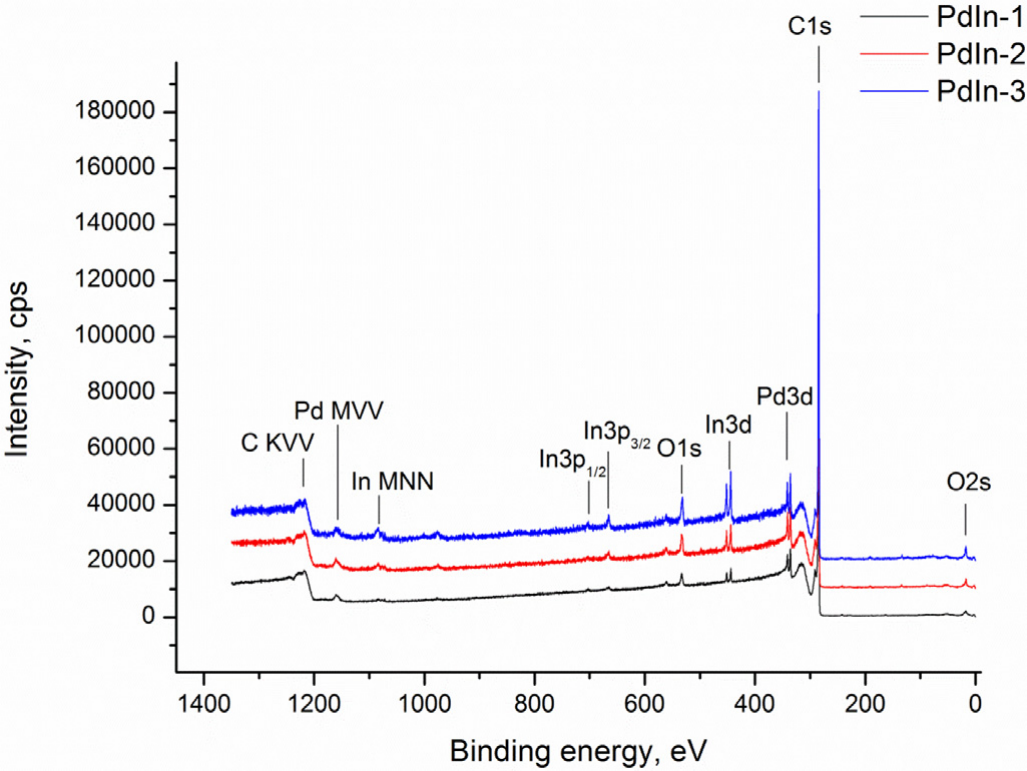
Survey XP spectra of the as prepared Pd-In/HOPG catalysts corresponding to different Pd:In ratios.

**Fig. 2:**
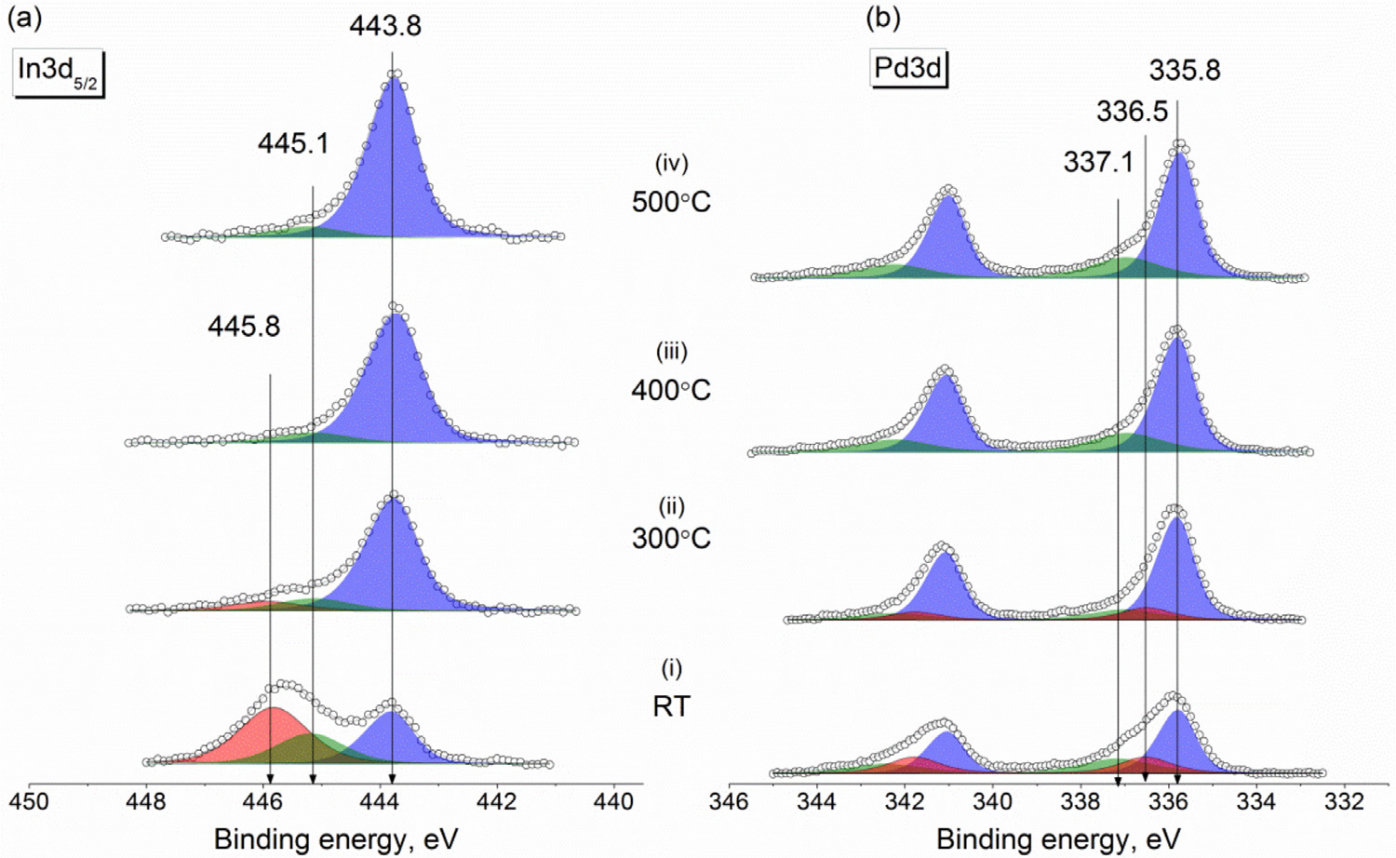
In3d_5/2_ (a) and Pd3d (b) core-level XP spectra of the PdIn-1 sample measured in UHV at room temperature for the pristine sample and those briefly annealed at 300 °C, 400 °C and 500 °C. The photoelectron kinetic energy of 300 eV used in the measurements corresponds to an effective probing depth of 2.4 nm.

**Fig. 3:**
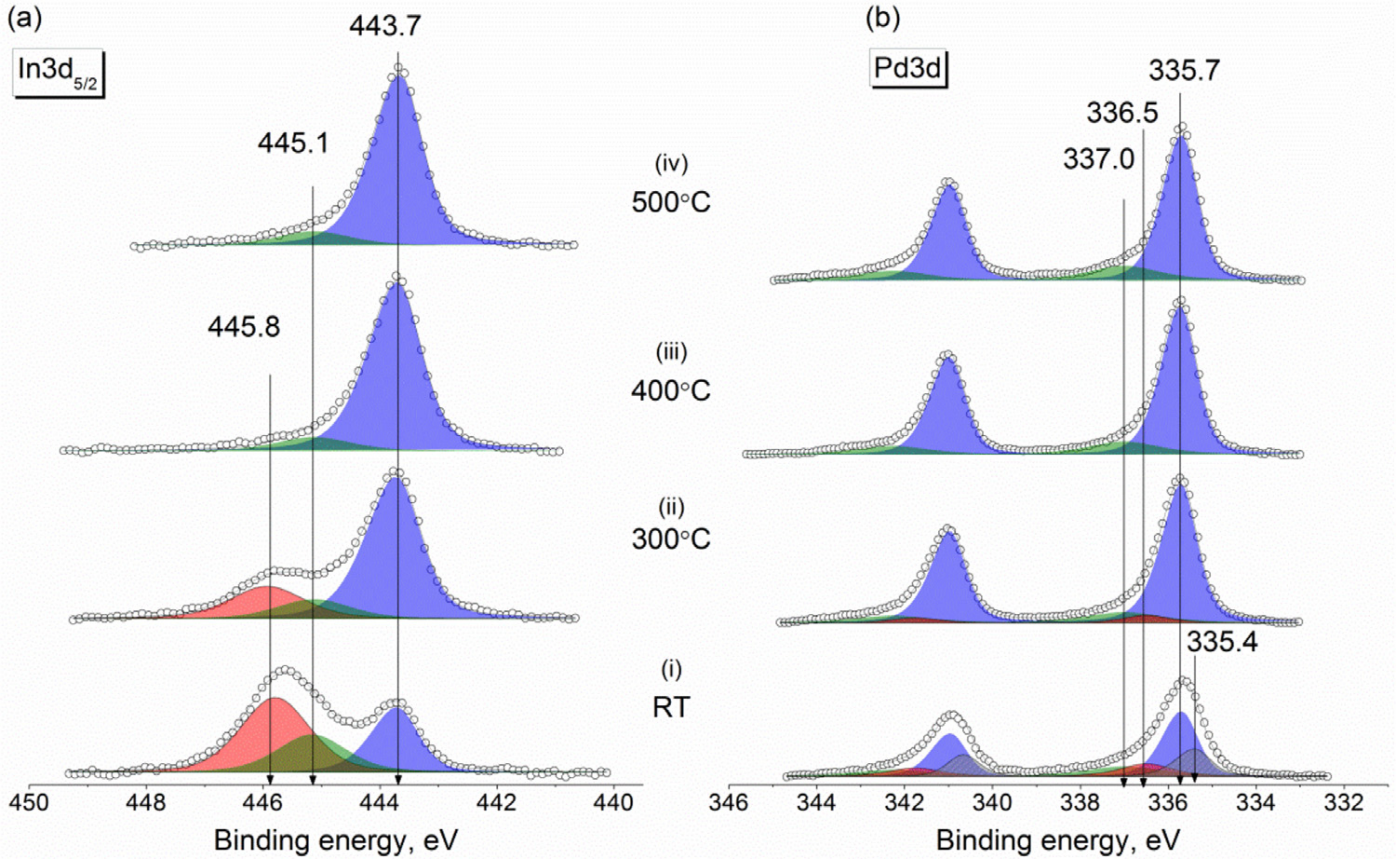
In3d_5/2_ (a) and Pd3d (b) core-level XP spectra of the PdIn-2 sample measured in UHV at room temperature for the pristine sample and those briefly annealed at 300 °C, 400 °C and 500 °C. The photoelectron kinetic energy of 300 eV used in the measurements corresponds to an effective probing depth of 2.4 nm.

**Fig. 4:**
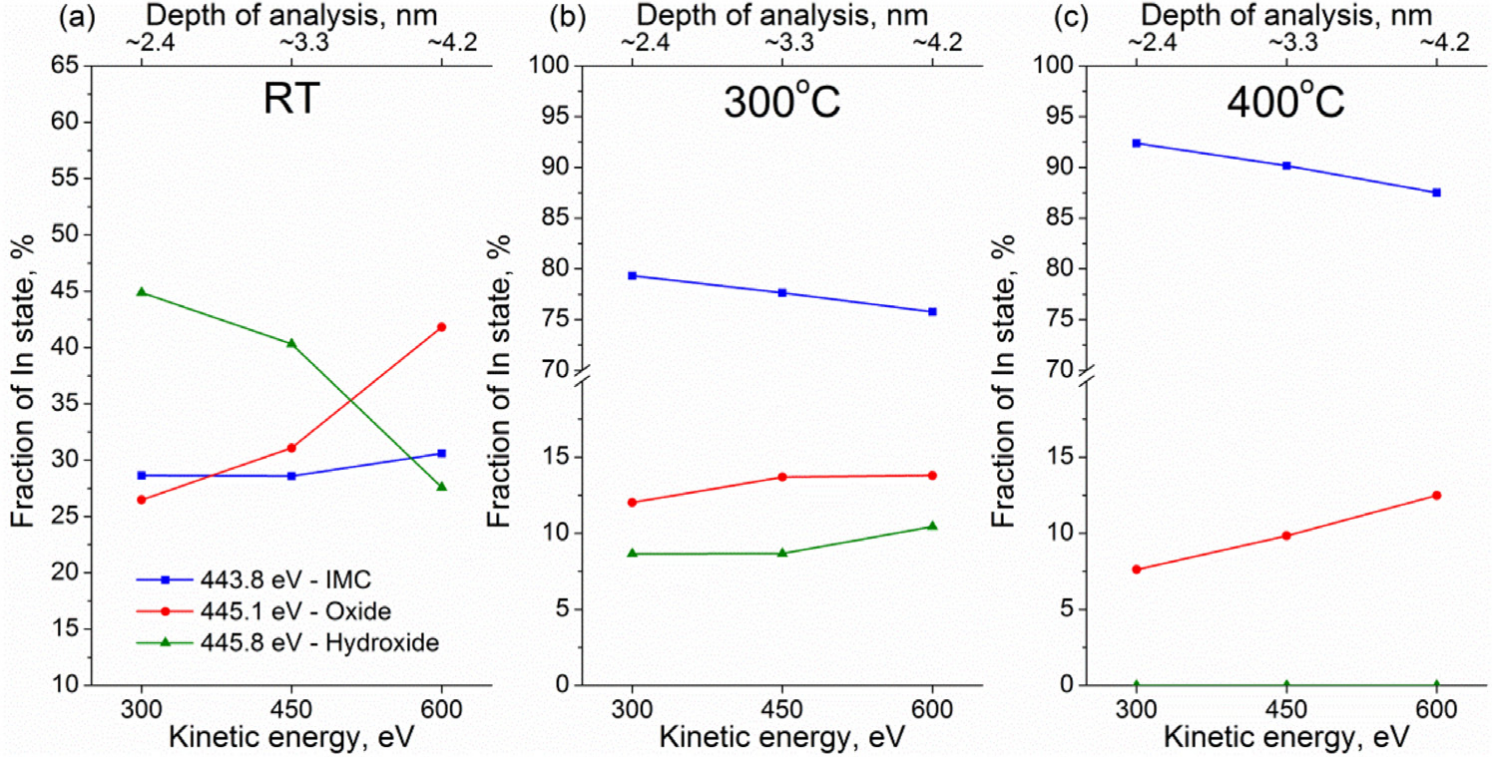
Fractions of chemically inequivalent In states as a function of photoelectron kinetic energy and effective depths of analysis derived from the In3d_5/2_ spectra measured at room temperature for PdIn-1: the as loaded sample (a), sample annealed in UHV at 300 °C (b) and at 400 °C (c).

**Fig. 5:**
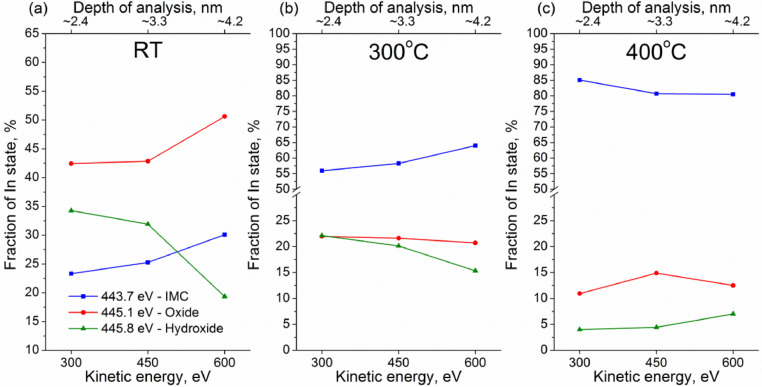
Fractions of chemically inequivalent In states as a function of photoelectron kinetic energy and effective depths of analysis derived from the In3d_5/2_ spectra measured at room temperature for PdIn-3: the as loaded sample (a), sample annealed in UHV at 300 °C (b) and at 400 °C (c).

**Fig. 6:**
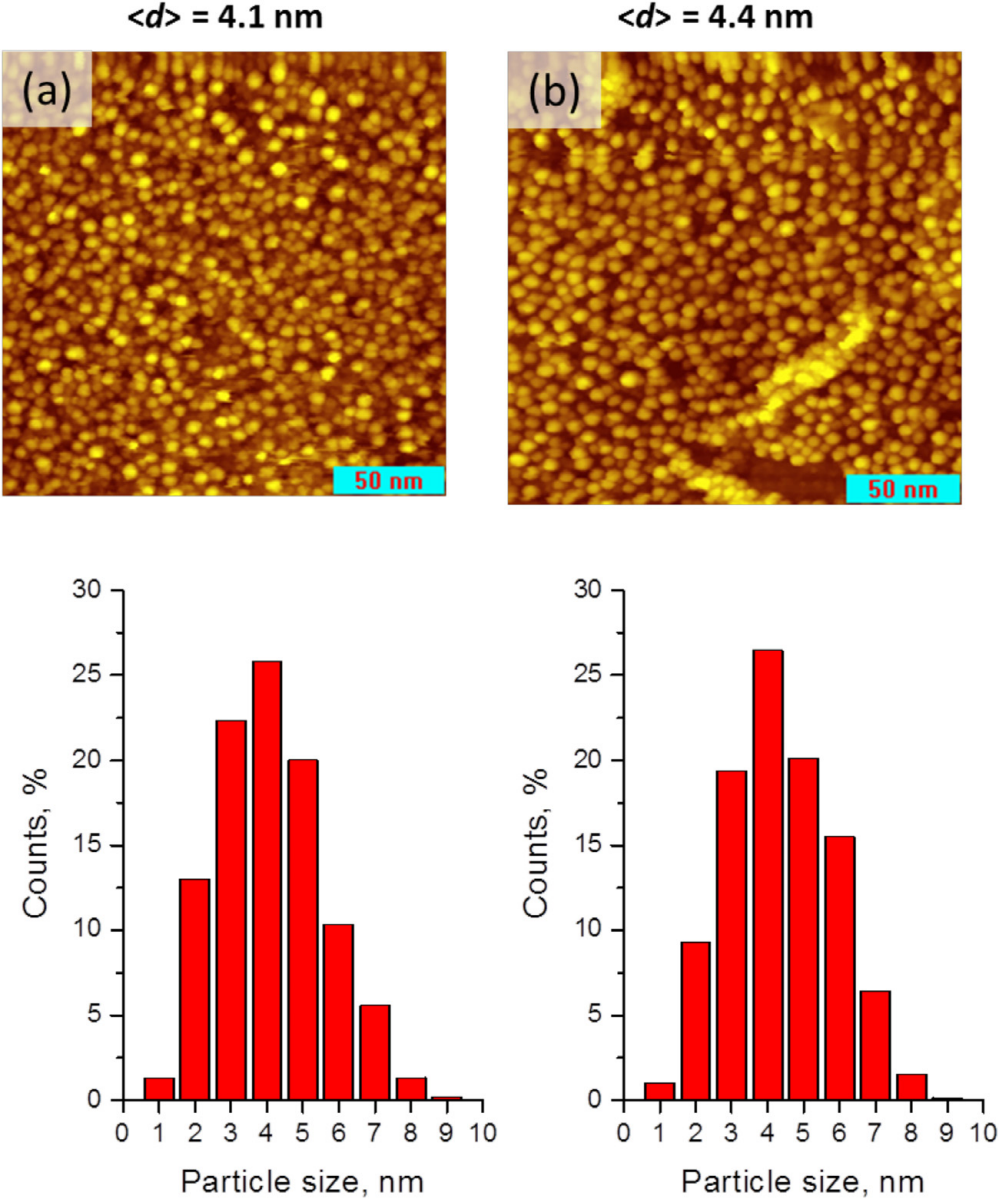
Raw STM images (field of view 200 × 200 nm) and essential morphological characteristics (particle size histogram and mean particle size) of PdIn-3 sample: (a) as prepared; (b) after O_2_-treatment. Tunneling parameters: (a) 0.47 nA, 1.50 V; (b) 0.50 nA, 1.50 V; The figure is modified from [1].

Experimental data discussed in the current article correspond to the samples described in the work «A mild post-synthesis oxidative treatment of Pd-In/HOPG bimetallic catalysts as a tool of their surface structure fine tuning», Appl.Surf.Sci [1]. Fig. 1 presents the survey XP spectra measured with a lab-based photoelectron spectrometer for the as prepared Pd-In/HOPG catalysts. Table 1 details exact parameters of the Omicron EFM3 evaporator (filament current, accelerating voltage, thermoelectron emission current and deposition duration) used for the metal deposition. In3d_5/2_ and Pd3d XP spectra for the PdIn-1 and PdIn-2 samples after their storage in air measured in UHV at different temperatures are presented in Figs. 2 and 3, respectively. These spectra correspond to the photoelectron kinetic energy of 300 eV; the experimental data were acquired at the Russian-German Laboratory beamline (RGL) at the HZB. The peak fitting parameters corresponding to this dataset are presented in Table 2, including binding energy, full width at half maximum (FWHM) and Gaussian-Lorentzian (GL) proportion of the profile sum function (where 0 corresponds to a pure Gaussian and 100 – to a pure Lorentzian). Fig. 4 plots trends in the distinct In state fractions as a function of the photoelectron kinetic energy corresponding to different effective analysis depths for the PdIn-1 sample in the as loaded form and ones after its heating to 300 °C and 400 °C in UHV. Meanwhile, Fig. 5 shows analogous data for the PdIn-3 samples. The peak fitting parameters for the Pd3d and In3d core-level lines are presented in Table 3. This dataset corresponds to XP spectra measured at the UE56/2 PGM-1 beamline. The raw STM images along with their derived particle size histograms and mean particles sizes for the as prepared and O_2_-treated PdIn-3 sample are shown in Fig. 6 which was modified from [1]. The raw data of information presented in each figure are uploaded on Mendeley Data (https://data.mendeley.com/datasets/2h773jgvbm/2).

**Table 1:** Exact parameters of the thermal vacuum deposition used for the set of Pd-In/HOPG samples under study.

Sample	Metal	Filament current, A	Accelerating voltage, V	Thermoelectron emission, mA	Time, min
PdIn-1	Pd	1.43	900	14.3–14.8	15
	In	1.33	500	4.3–4.4	2
PdIn-2	Pd	1.43	900	13.5–14.9	32
	In	1.33	500	4.4–4.5	3
PdIn-3	Pd	1.43	900	14.3–15.3	30
	In	1.33	500	4.4	5

**Table 2:** Peak fitting of Pd3d and In3d core-level spectra into individual components and their assignment (measurements at the RGL-beamline at HZB).

Pd3d_5/2_				In3d_5/2_			
Pd state	BE, eV	FWHM, eV	GL, %	In state	BE, eV	FWHM, eV	GL, %
Pd^0^	335.4–335.5	0.95–1.0	33–35	In_IMC_	443.7–444.0	0.97–1.1	35–40
Pd_IMC_	335.7–335.9	0.95–1.0	25–32	In_oxide_	445.1	1.3–1.4	40–42
Pd_oxide_	336.5	1.2–1.3	60	In_hydroxide_	445.8	1.3–1.4	40–45
Pd_clusters_	337.0-337.1	1.8–1.9	60	_–_

**Table 3:** Peak fitting of Pd3d and In3d core-level spectra into individual components and their assignment (measurements at the UE56/2 PGM-1 beamline at HZB).

Pd3d_5/2_				In3d_5/2_			
Pd state	BE, eV	FWHM, eV	GL, %	In state	BE, eV	FWHM, eV	GL, %
Pd^0^	335.5	0.82	34	In_IMC_	443.8–443.9	0.95–1.0	30–34
Pd_IMC_	335.8–335.9	0.87–0.92	34–36	In_oxide_	445.0	1.4	45–47
Pd_clusters_	337.0	1.7 –1.8	60	–

## 2 Experimental Design, Materials and Methods

The preparation of the Pd-In/HOPG samples has been carried out in a UHV chamber of a lab-based photoelecton spectrometer (SPECS, Germany) by a successive thermal vacuum metal deposition procedure as desrcibed elsewhere [2,3]. Small species of high purity (99.99) Pd and In foils have been loaded into a tantalum crucible and then evaporated on defected surface of HOPG using an Omicron EFM3 (Germany) electron beam evaporator. The sample was oriented horizontally inside the Preparation chamber and the evaporator was located at an angle of 90 °C to the surface of HOPG, distance between sample and evaporator was set to 15 mm. During the metal deposition the sample was kept at room temperature. The characterization of the prepared catalysts was performed using the same spectrometer in an analyzer chamber equpied with a hemispherical analyzer PHOIBOS-150-MCD-9, monochromator FOCUS 500 and an X-ray source XR 50M with double Al/Ag anodes. In the current work, AlKα radiation (hν = 1486.74 eV, 150 W) was used as X-ray source. The binding energy (BE) scale was calibrated using the positions of Au4f_7/2_ (84.0 eV), Ag3d_5/2_ (368.3 eV) and Cu2p_3/2_ (932.7 eV) from clean gold, silver and copper foils, respectively.

The IMC formation was studied by SRPES at the RGL-beamline at the Russian-German Laboratory (RGL) of the HZB [4]. The spectrometer was equipped with a hemispherical analyzer PHOIBOS-150-2D-CCD and an e-beam heating system which allowed us to heat the samples in the temperature range 50-1200 °C. The samples were stepwise heated in UHV from room temperature to 500 °C at 100 °C step and held at each temperature for 40 min. After each heating, the samples were cooled down, and XP spectra were measured in UHV at RT.

The effects exerted by the O_2_ treatment on the Pd-In/HOPG surface composition were studied with a NAP-XPS setup [5] at the UE56/2 PGM-1 beamline at the HZB. Annealing the samples was done by an infrared laser capable of heating in the temperature range 25-800 °C. The samples were annealed in UHV at 500 °C for 1 h to form Pd-In IMC and remove possible contaminations resulted from the sample storage in air. Then, 0.25 mbar O_2_ was fed into the analyzer chamber using the mass flow controller (Bronkhorst) followed by XP spectra measurements at room temperature, 150 °C and 200 °C.

To gather information about the depth distribution of the two metals, XP spectra were measured with synchrotron radiation-based XPS setups (both at the GRL-beamline and UE56/2 PGM-1) at three distinct photoelectron kinetic energies (300 eV, 450 eV, 600 eV) thus providing different effective probing depths. The apparent photoelectron inelastic mean-free paths (IMFP, λ) in Pd and In for these three energies were assumed to be 8 Å, 11 Å, and 14 Å, respectively, based on simulations using the QUASES-IMFP-TPP2M software [6] with the effective depths of analysis approximated as 3λ [7]. Raw XP spectra were corrected to the incident photon flux and quantitatively analyzed using photoionization cross-sections taken from [8]. Absolute positions of In3d and Pd3d peaks were calibrated against the C1s core level of HOPG (284.5 eV) measured at the same primary excitation energy. XP spectra were processed and fitted with individual components using the XPSPeak 4.1 software [9]. Shirley background has been subtracted from the In3d and Pd3d spectra before the fitting.

STM measurements of Pd-In/HOPG catalysts have been performed with a UHV 7000 VT microscope (RHK Technology, USA) operating in the constant current mode. Pt-Ir alloy cut wires with diameter of 0.25 mm were used as tips. Before measurements, STM images with atomic resolution of clean HOPG were recorded for the scanner calibration and also for control of the tip quality (achieving true atomic resolution was applied as a criterion of sufficient quality. The quantitative analysis of the experimental STM images processing was carried out by the web-based application ParticlesNN exploiting an advanced machine-learning particle recognition algorithm [10]. Mean particle sizes (<d>) were calculated by the following equation:

$$\langle d\rangle =\sum_{i}\left(d_{i}\times N_{i}\right)/\sum_{i}\left(N_{i}\right),$$

## CRediT Author Statement

**Maxim A. Panafidin:** Conceptualization, Investigation, Data curation, Writing – original draft, Writing – review & editing. **Andey V. Bukhtiyarov:** Conceptualization, Investigation, Data curation, Writing – original draft, Writing – review & editing; **Igor P. Prosvirin:** Investigation, Formal analysis, Writing – review & editing; **Igor A. Chetyrin:** Investigation, Formal analysis, Writing – review & editing; **Alexander Yu. Klyushin:** Investigation, Formal analysis, Writing – review & editing; **Axel Knop-Gericke:** Formal analysis, Writing – review & editing; **Nadezhda S. Smirnova:** Investigation, Formal analysis; **Pavel V. Markov:** Investigation, Formal analysis; **Igor S. Mashkovsky:** Investigation, Formal analysis; **Yan V. Zubavichus:** Writing – review & editing, Conceptualization, Supervision; **Aleksander Yu. Stakheev:** Writing – review & editing, Conceptualization, Supervision; **Valerii I. Bukhtiyarov:** Writing – review & editing, Conceptualization, Supervision.

## Declaration of Competing Interest

The authors declare that they have no known competing financial interests or personal relationships that could have appeared to influence the work reported in this paper.
